# Retrograde Intussusception and Giant Meckel’s Diverticulum: An Uncommon Encounter

**DOI:** 10.7759/cureus.25315

**Published:** 2022-05-25

**Authors:** Fiaz Ali, Rae-Ann Mohammed, Paige Ali, Lakhan Roop

**Affiliations:** 1 Paediatric Surgery, San Fernando Teaching Hospital, San Fernando, TTO; 2 Paediatrics and Child Health, San Fernando Teaching Hospital, San Fernando, TTO

**Keywords:** giant meckel’s diverticulum, meckel’s diverticulum, ileo-ileal intussusception, retrograde intussusception, paediatric intestinal obstruction

## Abstract

Retrograde intussusception (RINT) and giant Meckel’s diverticulum (MD) are both rare pathologies and are seldom encountered in surgical practice. Thus, it is exceptional for both conditions to be seen in the same patient, with very few published case reports in the paediatric population.

This case describes a three-month-old male who was referred to our paediatric surgery unit following a diagnosis of intussusception on an ultrasound scan. The patient presented to the paediatric emergency department one day prior with a clinical history of fever, cough, vomiting and irritability. After resuscitation, the patient was admitted for overnight observation in the paediatric ward. However, the patient’s symptoms persisted with notable abdominal distension. Abdominal X-ray (AP erect) showed features of small bowel obstruction, while abdominal ultrasound showed a concentric mass in the right upper quadrant consistent with intussusception. Following this diagnosis of intussusception, pneumatic enema reduction under ultrasound guidance was attempted but proved unsuccessful. The patient was then taken for emergency laparotomy. At surgery, an ileo-ileal RINT with a proximal giant MD was discovered. Successful manual reduction of the RINT and wedge resection of the giant MD with primary ileo-ileal anastomosis was performed. The postoperative recovery and follow-up were uneventful.

## Introduction

Intussusception is a common cause of intestinal obstruction in the paediatric population. However, retrograde intussusception (RINT) is a rare phenomenon, accounting for less than 1% of all intussusceptions [1]. Similarly, while Meckel’s diverticulum (MD) is the most common congenital anomaly of the gastrointestinal tract, giant MD is rare [2]. We present the case of a three-month-old male who presented with moderate grade respiratory and gastrointestinal symptoms. Due to persistent symptoms, radiological investigations were done which revealed intussusception. At laparotomy, the patient was found to have a RINT and a giant MD. Successful manual reduction of the RINT, decompression and resection of the giant MD, and bowel anastomosis were performed. No postoperative complications were encountered, and the patient remained symptom-free until discharge. To the best of our knowledge, and based on our literature search, this is the first reported case of both RINT and giant MD occurring simultaneously in a child in the Caribbean region. 

## Case presentation

A three-month-old infant presented to our paediatric emergency department with a one-day history of fever, cough, non-bilious vomiting, and irritability. On examination the patient had a benign abdomen with no palpable masses, and all blood investigations were within normal limits. After initial assessment, a diagnosis of viral gastroenteritis was made and the infant was admitted to the paediatric ward for intravenous rehydration therapy and overnight observation.

**Figure 1:**
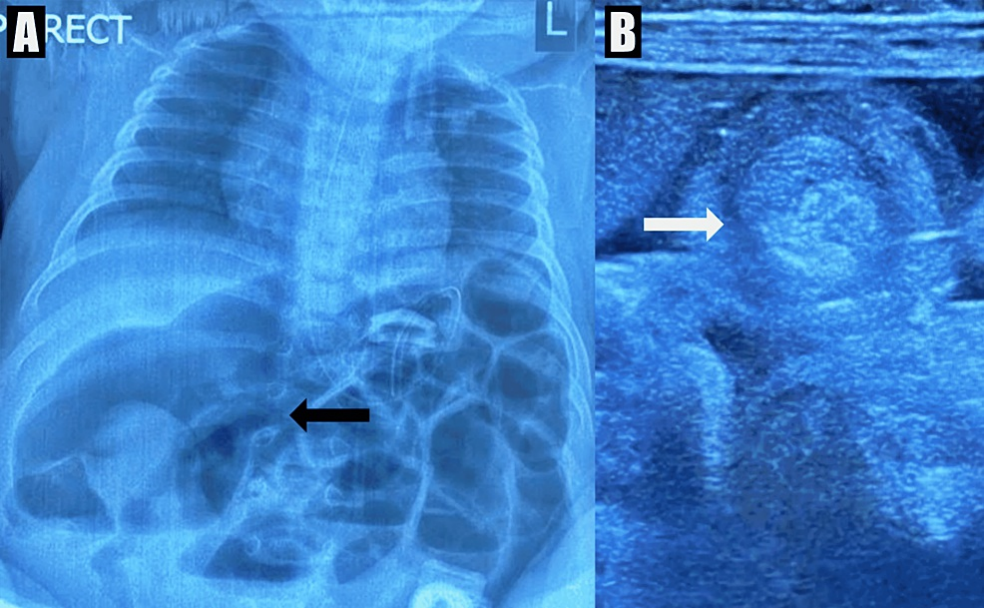
Preoperative radiological investigations (A) Erect abdominal X-ray showing features of small bowel obstruction with a very dilated segment of bowel in the right upper quadrant (black arrow). (B) Abdominal ultrasound scan showing a concentric mass (white arrow).

Throughout the night, the frequency of non-bilious vomiting gradually worsened. The following morning significant abdominal distension was noted, but there were no reported episodes of blood per rectum. Due to these findings, an abdominal X-ray (AP erect) and abdominal ultrasound scan were requested. Abdominal X-ray showed dilated loops of small bowel with multiple air fluid levels, and a significantly dilated segment of bowel located on the right side of the abdomen (Figure 1A). Abdominal ultrasound revealed a 2.1cm x 2.2cm concentric mass within the right upper quadrant of the abdomen (Figure 1B), with an approximately 10 cm segment of intussusception. Hence, a radiologic diagnosis of intussusception was made and ultrasound-guided pneumatic enema reduction was attempted once, but was unsuccessful. The patient was optimized appropriately, and subsequently taken for emergency laparotomy.

**Figure 2:**
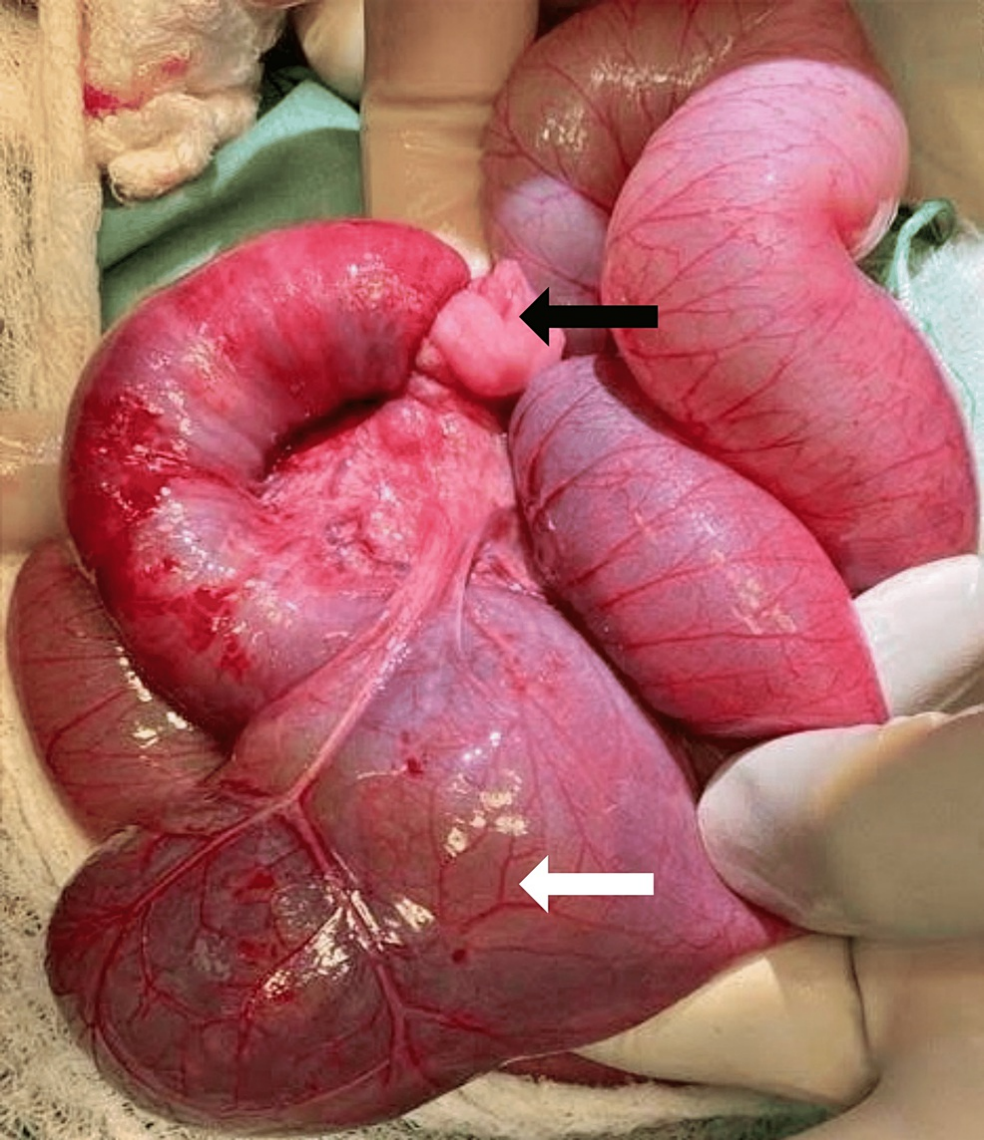
Intraoperative findings A segment of ileo-ileal retrograde intussusception (black arrow) and a giant Meckel’s diverticulum proximally (white arrow).

**Figure 3:**
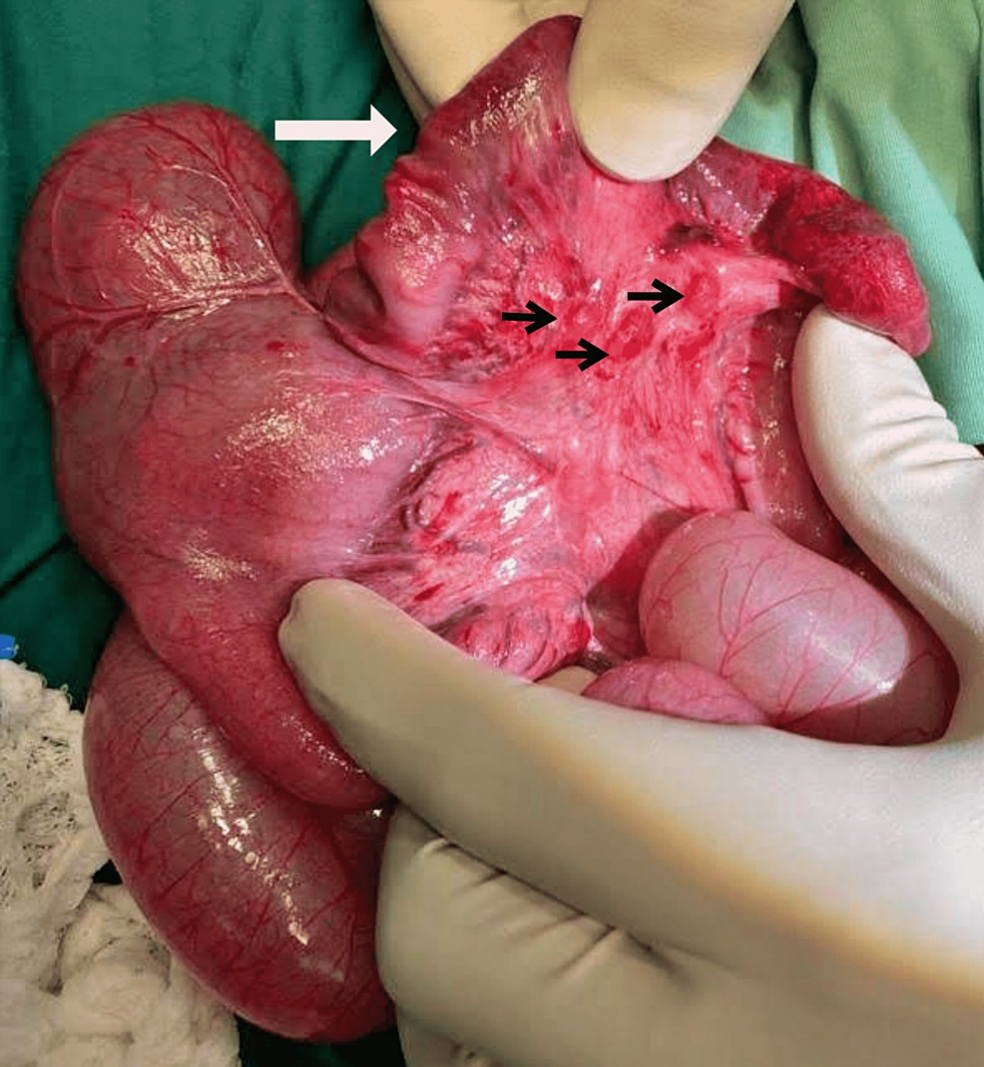
Post-reduction findings Viable small bowel on manual reduction of the retrograde intussusception (white arrow), and sub-centimetre mesenteric lymph nodes (black arrows).

A 15cm segment of ileo-ileal RINT was found at laparotomy, approximately 10cm from the ileocecal valve, with a giant MD located just proximal to this (Figure 2). The involved segment of ileum appeared healthy on manual reduction, with multiple sub-centimeter mesenteric lymph nodes (Figure 3). A giant MD with a base of approximately 10cm was found just proximal to the segment of intussusception, but not directly involved in the intussusception. A wedge resection of the giant MD and primary ileo-ileal anastomosis was successfully performed. 

Postoperatively, the patient was admitted to the Paediatric Surgical ward and had an unremarkable recovery. On the sixth postoperative day, he was discharged and subsequently followed up in the paediatric surgery outpatient clinic, with no complications to date. Histological analysis of the specimen did not reveal the presence of any heterotopic mucosa. 

## Discussion

Intussusception is defined as the telescoping of a proximal bowel segment into immediately adjacent distal bowel segment [3]. It is a common cause of intestinal obstruction in infants and young children. It is usually ileocolic and antegrade, with ileal lymphadenopathy including hypertrophic Peyer’s patches thought to be the lead point for ileocolic intussusception [4]. In 3% to 5% of cases, it may be associated with a MD [5]. 

Intussusception may be classified as antegrade or retrograde, with RINT being defined as the retrograde invagination of a distal intestinal segment into the lumen of the proximal intestine. RINT is far less common than antegrade, with a reported incidence in the paediatric population of approximately 0.2% [6]. However, it is increasingly being seen after Roux-en-Y gastric bypass surgery in adults, with an incidence of 0.1% to 0.3% [7]. It is thought that altered intestinal motility contributes to the development of RINT [8]. The RINT in our patient may have resulted from abnormal peristalsis in the ileum caused by the gastrointestinal infection. 

The preferred auxiliary investigation for intussusception is Colour Doppler Ultrasound (CDU), with a sensitivity of 97.5% and specificity of 99% [6]. However, due to its rarity RINT is seldom considered as a possibility when intussusception is found on CDU. Both hydrostatic and pneumatic enema reductions are the accepted methods of intussusception reduction currently. Pneumatic enema reduction under ultrasound guidance has been the chosen method of reduction at our institution for the past 20 years, with a success rate of greater than 90%. Nevertheless, both pneumatic and hydrostatic enema reductions are ineffective in RINT as they cause the mass of the intussusception to become pushed further inward, increasing the pressure and obstruction, and therefore leading to intestinal perforation [6]. The patient presented in this case report failed one attempt at pneumatic enema reduction and was taken for open surgical reduction that resulted in the discovery of a RINT.

Management includes manual reduction of the RINT intraoperatively with the possibility of resection of necrotic bowel and anastomosis, while the rest of the bowel is examined for other areas of intussusception. In the case of our patient, the involved segment of ileum was viable after manual reduction. However, a wedge resection and primary anastomosis was done due to the presence of a giant MD immediately proximal to the RINT.

MD is the most common congenital anomaly of the gastrointestinal tract. It is a true diverticulum as it contains all layers of the bowel wall and is derived from the incomplete obliteration of the vitellointestinal duct, which occurs around the fifth to seventh week of embryonic life. Histologically, it can also contain heterotopic mucosa, most commonly gastric or pancreatic in up to 20% to 50% of cases [2,9]. 

Giant MD is defined as diverticula measuring more than 5cm (two inches), which is an extremely rare entity in surgical practice. Limited cases have been reported in the paediatric population to date. While the majority of cases of MD are asymptomatic, the average incidence of symptomatic MD ranges from 4% to 9% for the general population, with approximately 60% of these cases present in the paediatric population. Furthermore, there is an inverse relationship between age and symptomatic presentation of MD, with an average of 50% of symptomatic patients being less than 10 years old. Several studies have also shown that only up to 5% of paediatric cases of symptomatic MD have been found to be giant MD at surgery, with a higher incidence of symptoms expected in such cases, which lends to the rarity of this case [2,9,10]. Complications of torsion and inflammation are more commonly seen with narrow-based, long MD while intestinal obstruction particularly by intussusception, is more frequently seen with wide-based, short MD [2,11,12].

The non-specific nature of the presenting symptoms of MD makes it difficult to diagnose particularly in the paediatric population, and oftentimes they are misdiagnosed most commonly as acute appendicitis. Only up to 4% of MDs are diagnosed preoperatively in patients who present with symptoms of acute abdomen. It still remains that the majority of cases are only given a conclusive diagnosis at surgery [9,13]. This was also the case in our patient as the giant MD was only discovered at laparotomy.

In children, resection of asymptomatic MD is generally not recommended due to the low risk of lifelong complications. Indications for resection include symptomatic MD, grossly abnormal appearance suggesting the presence of heterotopic mucosa, a narrow base of less than 2cm, and a giant MD, as was the case in our patient [2,9]. Surgical management of MD typically includes resection of the MD with possible resection of adjacent bowel, the extent of which is determined by the degree of inflammation seen at surgery [2]. Giant MD requires resection even if found incidentally at surgery given the high risk of more severe forms of complications, particularly obstruction, and low risk of morbidity associated with resection [11,14]. In the case presented, the decision was made to perform a wedge resection of the giant MD together with primary ileo-ileal anastomosis, based on the viability of the bowel seen after manual reduction of the RINT. The outcome of this procedure was successful and the patient has not experienced any complications to date.

## Conclusions

The occurrence of a giant MD together with RINT in this three-month-old infant highlights the unusual nature of this case. This case further emphasizes the possibility of MD in any patient presenting with acute abdomen. Given the high risk of associated complications, we recommend that giant MD should be excised even if asymptomatic, with wedge resection being a feasible surgical option. Furthermore, RINT can be considered as a possible diagnosis in failed enema reduction if the associated radiologic findings suggest the presence of a giant MD.
